# Computational Design of High Energy RDX-Based Derivatives: Property Prediction, Intermolecular Interactions, and Decomposition Mechanisms

**DOI:** 10.3390/molecules26237199

**Published:** 2021-11-27

**Authors:** Li Tang, Weihua Zhu

**Affiliations:** Institute for Computation in Molecular and Materials Science, School of Chemistry and Chemical Engineering, Nanjing University of Science and Technology, Nanjing 210094, China; tl609766532@126.com

**Keywords:** high energy compounds, property prediction, intermolecular interactions, decomposition mechanisms

## Abstract

A series of new high-energy insensitive compounds were designed based on 1,3,5-trinitro-1,3,5-triazinane (RDX) skeleton through incorporating -N(NO_2_)-CH_2_-N(NO_2_)-, -N(NH_2_)-, -N(NO_2_)-, and -O- linkages. Then, their electronic structures, heats of formation, detonation properties, and impact sensitivities were analyzed and predicted using DFT. The types of intermolecular interactions between their bimolecular assemble were analyzed. The thermal decomposition of one compound with excellent performance was studied through ab initio molecular dynamics simulations. All the designed compounds exhibit excellent detonation properties superior to 2,4,6,8,10,12-hexanitro-2,4,6,8,10,12-hexaazaisowurtzitane (CL-20), and lower impact sensitivity than CL-20. Thus, they may be viewed as promising candidates for high energy density compounds. Overall, our design strategy that the construction of bicyclic or cage compounds based on the RDX framework through incorporating the intermolecular linkages is very beneficial for developing novel energetic compounds with excellent detonation performance and low sensitivity.

## 1. Introduction

Energetic materials (EMs) are imperative parts in numerous areas due to their high energy. For example, they have applications in the military, mining, space, and other fields [1,2,3,4]. With the advancement of science and technology, there are continuing requirements for new EMs with fabulous performance and low sensitivity. Usually, a huge challenge is that the high energy and low sensitivity of EMs are mutually conditioned. Therefore, searching for new EMs with high-energy and insensitivity is in urgent need [5]. For this reason, the advancement of novel EMs has become one of the hot spots within the field of EMs.

**Figure 1:**
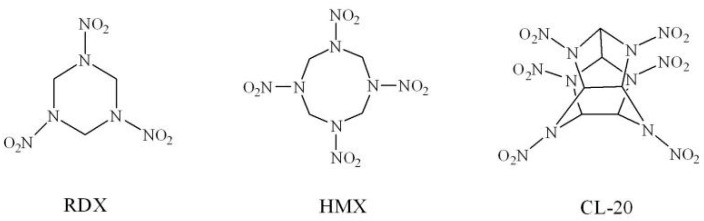
Molecular structures of RDX, HMX, and CL-20.

As the most promising class of compounds as high energy density compounds (HEDCs), cyclic compounds have been pulled in significant consideration due to their favorable energy and low sensitivity [5,6,7,8,9,10,11,12,13]. For instance, the two famous explosives 1,3,5-trinitro-1,3,5-triazinane, called RDX, and 1,3,5,7-tetranitro-1,3,5,7-tetrazocane, called HMX (Figure 1). They have been broadly utilized for military and civilian applications for a long time. Specifically, RDX has good detonation properties and low impact sensitivity. Its impact sensitivity (*h*_50_) is 26 cm, detonation velocity is 8.70 km/s, and detonation pressure is 34.0 GPa [14]. The detonation performance is exceptionally near to the standard of HEDCs, whose detonation velocity is more than 9.0 km/s and detonation pressure is more than 40.0 GPa [15,16]. Accordingly, the skeleton of RDX could be regarded as a skeleton structure for the improvement of HEDCs.

It is found that joining nitro groups by means of C- or N-functionalization in the RDX skeleton may be a well-known and effective technique for achieving better energetic performances. At present, some new cyclic compounds have been successfully created as the potential candidates of HEDCs. Unfortunately, when these derivatives have more nitro groups, their energy performance has been greatly improved, but at the same time this also makes them very sensitive to external stimuli and very dangerous in experiments and industry. Furthermore, energetic compounds containing more nitro groups are difficult to be synthesized. Hence, further study to find new ways to design new HEDCs based on the RDX structure is needed.

In general, compared to monocyclic compounds, polycyclic and cage compounds such as 2,4,6,8,10,12-hexanitro-2,4,6,8,10,12-hexaazaisowurtzitane (CL-20, Figure 1), etc. have huge ring or cage strain [4,17]. So, the polycyclic and cage compounds may have higher detonation properties. Pan et al. [18] reported a new method to build bicyclic and cage compounds on the skeleton of HMX and found that these novel candidates of HEDCs have both fabulous performance and low sensitivity. Therefore, building polycyclic and cage structures based on the system of RDX might lead to surprising increase in the detonation performance. Additionally, due to incomplete redox reaction, the low oxygen balance (OB) of RDX (−21.61%) leads to a lower amount of energy released. Of course, the introduction of nitro groups into newly designed RDX-based compounds to improve oxygen balance is also essential.

Within the design of HEDCs, their crystal structure must be considered [19]. However, the designed HEDCs have not been synthesized, so their crystal structure cannot be decided by tests. Therefore, it is very important to predict the crystal structure of designed HEDCs. Johnson et al. [20] demonstrated that the RDG strategy is a viable method to show the interactions, such as hydrogen bond, van der Waals force, and steric effect. Meanwhile, it is well-known that data on the decomposition process of the explosives are valuable to understand the behaviors. Recently, ab initio molecular dynamics (AIMD) strategy has been effectively employed to investigate the decomposition mechanisms [21,22]. Additionally, these studies give some valuable data to understand the decomposition process of the explosives at high temperature. For example, Xiang et al. [23] investigated the decomposition process of the 4,10-dinitro-2,6,8,12-tetraoxa-4,10-diazaisowurtzitane (TEX) crystal at high temperature of 2160 K by AIMD simulations. Wu et al. [24] performed AIMD simulations to examine the decomposition of 3,6-di(azido)-1,2,4,5-tetrazine (DiAT) at 3000 K. In spite of the fact that this research give important information to understand the decomposition reactions of the explosives, a comprehensive understanding of their decomposition process is still unclear.

**Figure 2:**
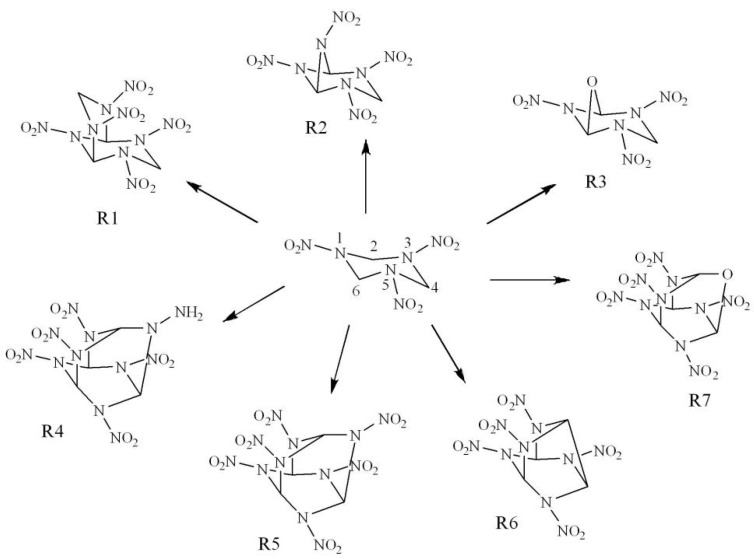
Molecular frameworks of seven designed compounds (R1−R7).

In this work, seven novel high energy compounds (Figure 2) were designed using a new strategy. To begin, -N(NO_2_)-CH_2_-N(NO_2_)- and -N(NO_2_)- were selected as intermolecular linkages rather than substituents. At the same time, the -O- linkage can improve the oxygen balance effectively. In addition, three new bicyclic compounds R1, R2, and R3 were generated by connecting the C2 and C6 (or C4) atoms in the RDX structure using intermolecular linkages. After that, four new cage compounds were produced by adding -N(NH_2_)-, -N(NO_2_)-, and -O- linkages to connect the C atoms within the molecular structure of the bicyclic compounds. Then, their electronic structures, heats of formation (HOFs), energetic properties, OBs, and impact sensitivities of the designed compounds were studied carefully using density functional theory (DFT). The interaction energy, nitro charge change, electron density topology, reduction density gradient, and electron density difference analysis were considered to analyze the interactions between biomolecules and to discuss the stability of these high energy compounds. Their crystals were predicted by Polymorph and then the beginning chemical processes of the crystal of R3 at high temperature of 2500 K was simulated by AIMD. Our fundamental aim is to look for new high-energy insensitive energetic compounds.

## 2. Results and Discussion

### 2.1. Property Prediction

#### 2.1.1. Molecular Geometry and Electronic Structures

**Figure 3:**
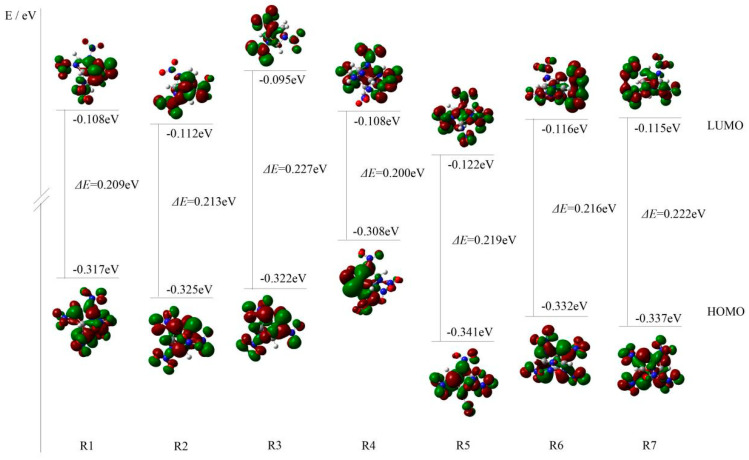
HOMO and LUMO energy levels and energy gaps of seven designed compounds.

There are two important orbitals in molecular orbitals, the highest occupied molecular orbital (HOMO) and lowest unoccupied molecular orbital (LUMO). Additionally, their energy gaps (Δ*E*) can reflect the activity. It was reported that B3LYP is an effective method used to calculate the energy gaps of the energetic compounds and can give reliable qualitative variation trend for the energy gaps [6,18], so the energy gaps for the designed compounds were calculated at this level. Figure 3 shows the 3D plots of HOMO, LUMO, and Δ*E* of the seven designed compounds, where the positive phase was appeared in red area and the negative was appeared in green area. As shown in Figure 3, all the designed compounds have energy gaps ranging from 0.200 to 0.227 eV. Additionally, R3 has the biggest energy gap, whereas R4 has the smallest one. This is due to large differences in the molecular structures between R3 and R4. R3 has smaller cage with fewer nitro groups, whereas R4 has bigger cage with more nitro groups.

**Figure 4:**
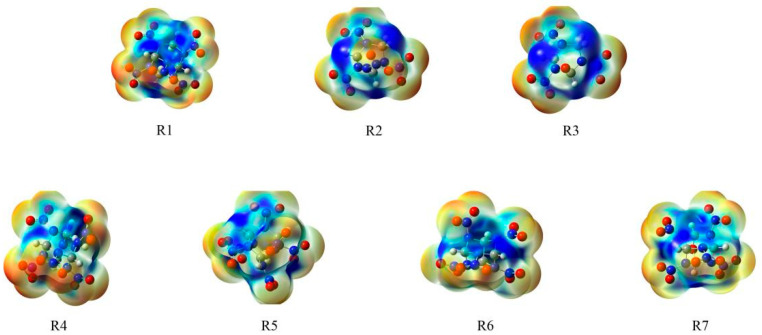
Electrostatic potentials mapped of the designed compounds. Color coding for MEPs range from red (negative) to blue (positive).

Previous studies showed that the energetic compounds are more sensitive when they have greater positive potential in the molecules [25]. Figure 4 outlines the molecular electrostatic potentials (MEPs) of the 0.001 electron/bohr^3^ electron density isosurface of the title compounds. The red color represents negative potential and blue signifies positive potential with the range from −0.03 to +0.05 hartree. Based on the MEPs of the designed compounds, the positive potentials are at the center of the bicyclic or cage, whereas the negative potentials locate on the NO_2_. Among these compounds, R4, R5, R6, and R7 have more positive potential than the other three compounds, which may lead to higher sensitivity.

#### 2.1.2. HOFs

To estimate the detonation velocity and pressure by theoretical calculations, the value of Δ*H*_f_ needs to be found first. However, it is difficult to measure Δ*H*_f_ through tests, so theoretical strategy is used to predict Δ*H*_f_ by means of isodesmic reactions. Table S1 of Supporting Information lists the total energies (*E*_0_), zero-point energies (*ZPE*), thermal correction (*H*_T_), and HOFs of the reference compounds within the isodesmic reactions.

Table S2 of Supporting Information lists the total energies, *ZPE*s, thermal corrections, gas-phase HOFs, heat of sublimation, and solid-phase HOFs of the designed compounds. Clearly, all the designed compounds show highly positive gas and solid-phase HOFs. Among them, the gas and solid phase HOFs of R5 are the highest, which are 1556.46 kJ/mol and 1423.97 kJ/mol, respectively. It can be seen that the design of bicyclic or cage compounds by combining high-energy groups can significantly increase the HOFs. This is as the inherent strain existing in bicyclic or cage compounds can release more energy during combustion [4,26].

#### 2.1.3. Detonation Properties

Table 1 lists the density (*ρ*), heat of detonation (*Q*), detonation velocity (*D*), detonation pressure (*P*) and oxygen balance (OB) of the designed compounds. All the designed compounds, save R1, have higher densities than RDX. Among them, R4, R5, R6, and R7 have higher densities than HMX. However, these compounds have lower densities than CL-20. Furthermore, R5 and R7 have the highest density (1.98 g/cm^3^) as they have the foremost nitro groups.

As appeared in Table 1, the OB values of all the compounds are in the range from –6.78% to +7.77%, which are higher than those of RDX, HMX, and CL-20, whose values are −21.62%, −21.62%, and −10.96%, respectively. In particular, the OB values of R2 and R6 rise to zero, making them possess excellent detonation properties as the energy is discharged completely within the explosion. Additionally, all the designed compounds have higher *Q* than their parent compound RDX. Among them, R2 has an extraordinarily high *Q* value of 2291.20 cal/g.

The detonation velocities of the designed compounds vary from 9.63 to 10.27 km/s, which are much higher than those of RDX, HMX, and CL-20, whose values are 8.70 km/s, 9.10 km/s, and 9.40 km/s, respectively. Additionally, R6 has the highest detonation velocity of 10.27 km/s among these compounds, which surpasses that of CL-20. The detonation pressures of the designed compounds are from 40.86 to 48.91 GPa, much higher than those of RDX, HMX, and CL-20, which are 34.00 GPa, 39.30 GPa, and 42.00 GPa, respectively. R6 has the highest detonation pressures of 48.91 GPa among the designed compounds, over that of CL-20. In addition, the -O- linkage within the compounds R3 and R7 decreases the detonation velocity and detonation pressure altogether. Along with the increasing numbers of the nitro groups, the detonation velocities and pressures of the designed compounds improve. Overall, all the designed compounds possess surprisingly high detonation performances over RDX, HMX, and CL-20 due to the bicycle and cage structure within the molecules. Therefore, the construction of the bicyclic or cage compounds based on the RDX system can successfully improve energy properties.

#### 2.1.4. Impact Sensitivity

Impact sensitivity (*h*_50_) is an index to evaluate the sensitivity of energetic compounds [27]. In most cases, the higher *h*_50_ is, the more stable the explosive is.

As seen in Table 1, all designed compounds have higher *h*_50_ values than CL-20, lower than the parent compound RDX but for R1 and R3, and lower than HMX but for R1 and R3. It can be concluded that R1–R7 are more stable than CL-20. Among them, R1 and R3 are more insensitive to external impact than RDX and HMX. In particular, R3 has the highest *h*_50_ value of 40.73 cm. Subsequently, the joining of the oxygen linkage can successfully decrease the sensitivity of the bicyclic and cage compounds. This is consistent with the results of the previous molecular electrostatic potential analysis.

Overall, all the designed compounds are regarded as potential candidates of HEDCs. In particular, R1 and R3 display fabulous detonation properties superior to RDX, HMX, and comparable to CL-20, and lower impact sensitivity than RDX and HMX. Consequently, R1 and R3 may be exceptionally appealing potential HEDCs.

### 2.2. Molecular Packing

In order to determine the molecular packing of the designed compounds, 10 space groups which account for nearly 86.49% of organic molecular solids are mainly considered. They are *P*2_1_/*c* of 36.59%, $P\bar{1}$ of 16.92%, *P*2_1_2_1_2_1_ of 11.00%, *C*2/*c* of 6.95%, *P*2_1_ of 6.35%, *Pbca* of 4.24%, *Pna*2_1_ of 1.63%, *CC* of 0.9%, *C*_2_ of 0.9%, and *Pbcn* of 1.01% [28,29]. These ten possible space groups are sufficient to find the most stable polymorphs for organic compounds. The energies and densities of the packing structures within the ten most common space groups are shown in Figure S1.

Seen from Figure S1, R1 and R6 are anticipated to have the *CC* space group for the possible packing, with the lowest energy and highest crystal density. R3, R4, and R5 have the $P\bar{1}$ space group for the most possible packing. R2 has the *Pna*2_1_ space group, while R7 has the *P*2_1_2_1_2_1_ space group.

### 2.3. Intermolecular Interactions

In this section, we will study the intermolecular interactions in the bimolecular assembles for the 7 designed compounds R1–R7 to estimate their stability.

#### 2.3.1. Geometrical Structures

The optimized structures of the bimolecular assembles by using B3LYP/6-311G (d, p) are displayed in Figure S2 of Supporting Information. It is seen in Figure S2 that the intermolecular interactions of the bimolecular assembles mainly exist among the H, N, and O atoms. 

Table 2 lists the distances of the most intermolecular interactions for the bimolecular assembles. For the intermolecular interactions between H···O, the distances are in the range of 2.44~2.86 Å except for R6 and R7, belonging to the hydrogen bonding distance. The shorter the distance is, the stronger the hydrogen bonding between O and H atoms is. Thus, there are extensive hydrogen bonding interactions in the bimolecular assembles, but the interactions in R2 and R3 are stronger.

#### 2.3.2. Interaction Energy Analysis

The intermolecular interaction energies *E*(int.) of seven bimolecular assembles were calculated at the B3LYP/6-311G (d, p) level. The results were corrected by basis set superposition error (BSSE) using counterpoise correction (CP). The intermolecular interaction energies *E*(int.) of seven bimolecular assembles are listed in Table 3. 

The interaction energies of the seven bimolecular assembles increase in the order of R3 < R1 < R2 < R5 < R6 < R7 < R4. For the most part, the smaller the interaction energy is, the more stable the assemble is. Therefore, their stability position is R3 > R1 > R2 > R5 > R6 > R7 > R4. There is hydrogen bonding within the intermolecular interactions in the bimolecular assembles. This is the same as the conclusion drawn from the impact sensitivity that R3 is most stable in these assembles.

#### 2.3.3. AIM Topological Analysis

AIM topological analysis at the basic point of the bond is used to examine the interactions between bimolecule assembles and to analyze the interaction [30]. The total electron density *ρ*(r) indicates hydrogen bond effect. The Laplace values of electron density ∇^2^*ρ*(r) and energy density *H*(r) can reflect the type of intermolecular interactions, where the energy density *H*(r) is equal to the whole of the potential energy density *V*(r) and Lagrangian kinetic energy density *G*(r) when the Laplace values of electron density and energy density are negative for the covalent bond interactions but are positive for the closed shell interactions (hydrogen bond, van der Waals interaction, etc.).

As shown in Table 2, *ρ*(r) is within the range of 0.00079~0.009488 a.u. ∇^2^*ρ*(r) and *H*(r) are both positive. This means that there are closed shell interactions in the bimolecule assembles. It is found based on the *ρ*(r) values of the seven assembles that the hydrogen bonding in R1, R2, and R3 is stronger. In addition, the interactions between O···O and O···N still exist in the bimolecule compounds, and their intensity is less than that of the hydrogen bonding. Therefore, the weak hydrogen bonding and van der Waals interactions exist in the bimolecule assembles.

#### 2.3.4. Reduced Density Gradients

**Figure 5:**
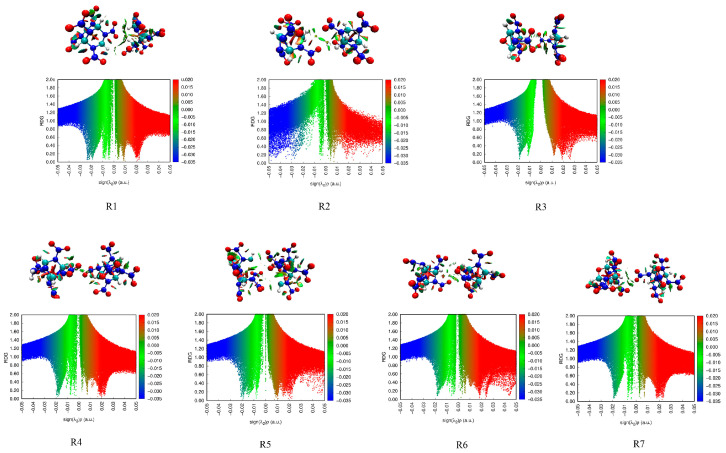
Scatter diagram and isosurface graph of RDG for configurations of seven bimolecular assembles.

The reduced density gradients (RDG) of these seven bimolecular assembles were analyzed in Figure 5. The scatter plot and filled isosurface plots can visually analyze weak interactions. The denser the points in the scatter diagram are, the greater the electron density is, and the stronger the effect is. Within the isosurface filling map, different colored areas represent different kinds of interactions. The blue area represents a strong attraction, such as a hydrogen bond or strong halogen bond, the green area is van der Waals force, and the red area is strong repulsion, such as steric effect.

In Figure 5, the two peaks of sign(*λ*_2_)*ρ* in the range of −0.03~0 a.u. indicate two types of interactions in the molecules: weak hydrogen bonding (−0.03~−0.01 a.u.) and van der Waals interaction (−0.01~0 a.u.). In addition, hydrogen bonding is the main intermolecular force. The values of sign(*λ*_2_)*ρ* are over 0, so there is a steric effect and the red area is shown in the bimolecular assembles. For R1, R2, and R3, the dots on the graph are denser than those of other compounds, indicating that there are stronger weak hydrogen bonding and van der Waals interactions. This is the same as previous conclusions that R1, R2, and R3 are more stable than R4−R7. Within the RDG color-filled maps, there are green areas in the oxygen atoms of the nitro groups and the hydrogen atoms, showing weak hydrogen bonds and van der Waals interactions in the bimolecular assembles. In R2, R3, R6, and R7, the light green regions appear between the oxygen atoms of the nitro groups in the bimolecular assembles, indicating the existence of O···O interactions.

#### 2.3.5. Electron Density Difference

The difference in electron density can intuitively describe the change in density between some atoms after the formation of the bimolecular assemble, showing the weakening and enhancement of corresponding bonds [31]. 

**Figure 6:**
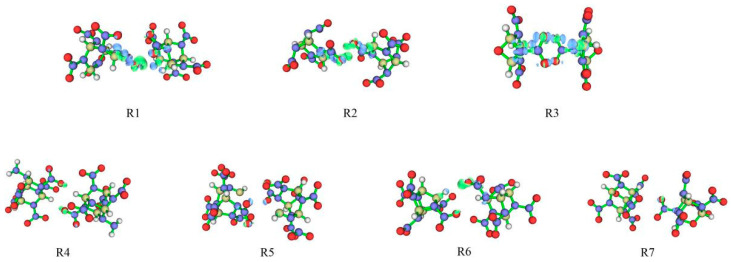
Electron density differences of seven bimolecular assembles.

In Figure 6, green and blue correspond to the density difference in isosurfaces of 0.001 and −0.001, respectively, showing the main areas where the electron density increases or decreases. Due to the hydrogen bond and van der Waals interactions, the oxygen atom area shows green, and hydrogen atom presents blue, confirming that the electron density of the hydrogen atom is reduced when the hydrogen bond is formed. The green area near part of the nitro group indicates the transfer of electrons from the hydrogen atom to the initiation bond, thereby increasing the strength of the initiation bond. It can be found that R3 is the most stable due to the larger density difference, followed by R1, R2, and R4–R7.

### 2.4. Thermal Decomposition

Among the seven designed compounds, R3 has excellent detonation performance and lowest impact sensitivity. Therefore, we will stimulate its decomposition process at a high temperature of 2500 K.

#### 2.4.1. Initial Decomposition Mechanisms

**Figure 7:**
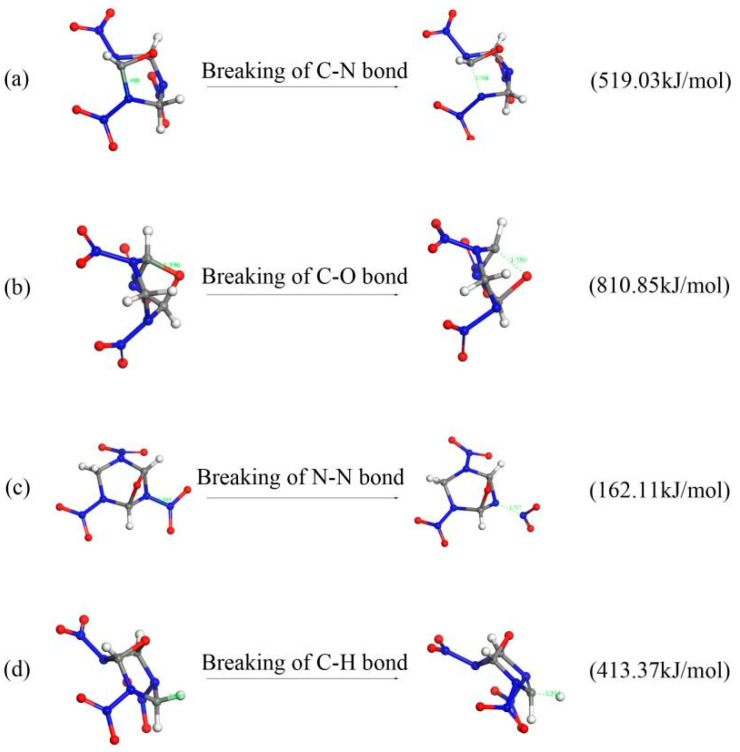
Four different initiation reactions in the thermal decomposition of the R3 crystal.

There are four different initiation reactions during the thermal decomposition of the R3 crystal. As shown in Figure 7, (a) the carbon-nitrogen ring was broken through C-N cleavage. The C-N bond length increases from 1.468 to 1.768 Å. This reaction occurs at 0.008 ps of the decomposition. (b) The oxygen ring was opened by breaking the C-O bond. The C-O bond length increases from 1.396 to 1.750 Å. (c) The N-NO_2_ bond broke and released NO_2_, and the bond length increases from 1.364 to 1.717 Å. (d) The breaking of the C-H bond released H radical, and the bond length changes from 1.092 to 1.314 Å.

In order to better understand the initial decomposition, the bond dissociation energy (BDE) of these initial reactions were further analyzed by DFT calculations. According to the DFT calculated results in Figure 7, the BDE of N-NO_2_ is 162.11 kJ/mol and is the smallest. This is the reason that the N-NO_2_ bond is first to decompose.

#### 2.4.2. Subsequent Decomposition

Four initial reactions promoted consequent decomposition of the R3 crystal together. Particularly, the H radical in path (d) could directly attack other parts and lead to their decomposition. Xiang et al. [23] explained in detail that the H radicals are very active and may cause other reactions. This is consistent with our finding in the decomposition of the R3 crystal.

**Figure 8:**
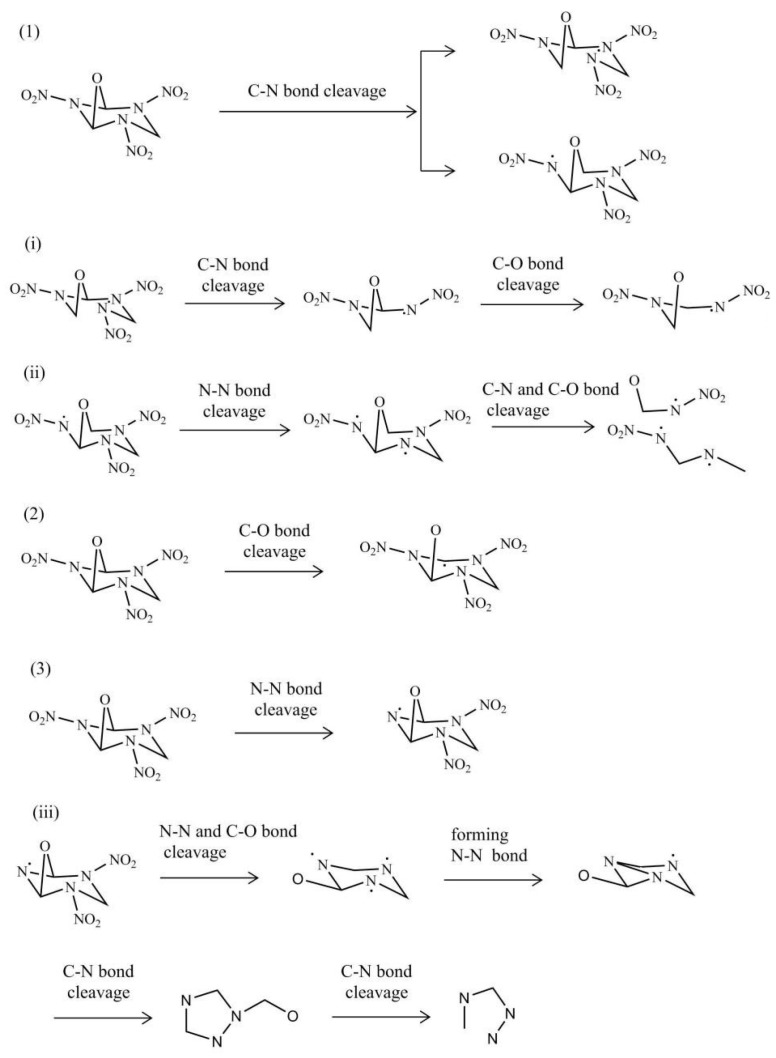
Subsequent decomposition process of the R3 crystal.

Different initial decomposition reactions result in different subsequent decompositions. More detailed decomposition processes happened after the four initiation reactions were displayed in Figure 8. It is found that the cage-opening mechanisms of the molecules are different: (1) The H radical attacked the C-N bond to open the cage structure. As more than one kind of C–N bond were broken, some isomers were produced. (2) The H radical attacked the C-O bond and then opens the cage structure. The cage breaking by the C-O bond cleavage could form the six-membered ring. After the cage structure was destroyed, the C-O and C-N bonds were broken to form long chains and release NO_2_. All the long chains are very unstable and rapidly decomposed into small radicals. (3) The H radical attacked the N atoms within the cage and caused the N-NO_2_ bond to crack. The catalytic behavior of the hydrogen radical is similar to that described in previous reports on the thermal decomposition of crystalline TEX [23] and furoxan [24] by AIMD simulations. Therefore, our results can provide information for a comprehensive understanding of the thermal decomposition process for the energetic materials.

For the subsequent decomposition process, there are two kinds of reaction paths. One is that long chains gradually decomposed into small radicals. Another is to form a new ring after the ring was opened, which was broken to a certain content. The long chain has undergone a rearrangement to break down into small molecules, such as NO_2_, NO, and CO. The snapshots of the decomposition mechanism (i) were displayed in Figure S3 of Supporting Information. Figure S4 of Supporting Information shows the snapshots of the decomposition mechanism (iii). After the six-membered ring was formed by the C-O bond cleavage, the new cage was produced by forming a new N-N bond. Through a series of bond cleavage and recombination reactions, a new cage was formed (Figure S4b). Afterwards, the new cage was broken, and the long chain decomposed into small fragments.

#### 2.4.3. Decomposition Products

**Figure 9:**
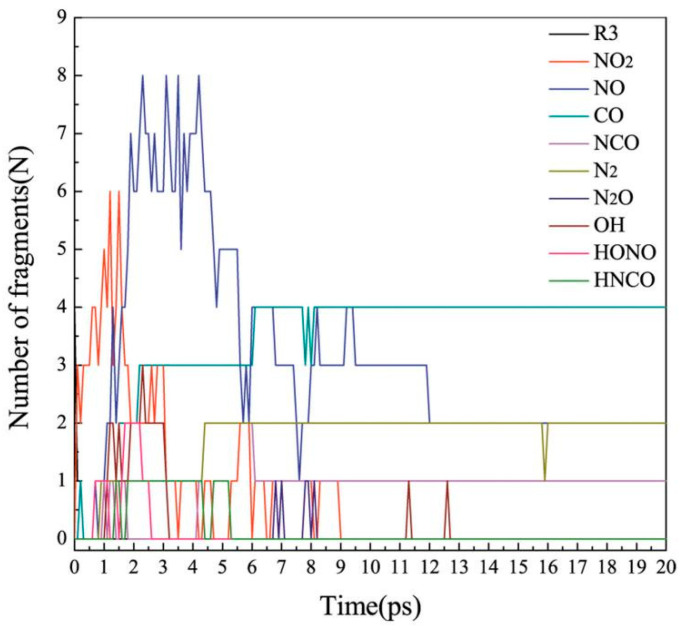
Time evolution of the number of main intermediates and products during the decomposition of the R3 crystal.

To achieve full decomposition process intuitively, Figure 9 shows the changes in the number of main intermediates and products over time during the decomposition of the R3 crystal. As shown in Figure 9, the molecule in the R3 crystal has decomposed completely after 0.25 ps. It shows that the decomposition rate of the crystal is very fast at a temperature of 2500 K.

Seen from Figure 9, the whole decomposition process of the R3 crystal could be roughly divided into two stages. We chose 9 ps as a critical point for the decomposition of the R3 crystal. The decomposition before 9 ps is a main decomposition process, whereas that after 9 ps is a stable stage. Before 9 ps, NO_2_ and NO were the main decomposition products, following the mechanisms mentioned above. When the C-NO_2_ and N-NO_2_ bonds were broken, the amount of NO_2_ reached a maximum at about 1.5 ps, then decreased steadily, and finally disappeared after 9 ps. At this time, the amount of NO increased sharply. Most NO were produced by the N-O bond breaking in NO_2_. Similarly, the amount of NO gradually decreased after reaching the maximum and stabilized at around 2 molecules after 9 ps. NO existed longer than NO_2_. As we know, CO and N_2_ are the main products formed in the decomposition process. At the beginning, the number of the CO molecule increased and finally reached around 4 molecules after 6 ps. The number of N_2_ started to increase after 0.9 ps. At 4.4 ps, the amount of N_2_ reached a stable value. This stable amount of N_2_ and CO lasted in the remaining time. We also note that the change in the amount of N_2_O is related to the amount of N_2_. When the concentration of N_2_ reached a stable value, the amount of N_2_O began to decrease. This also proves that most of N_2_ came from N_2_O.

In the whole decomposition process, although some small molecules are not high in concentration, they cannot be ignored. NCO was formed after 4.2 ps and after that its concentration reached around to 0–4 molecules. OH was produced at 1.1 ps, then its concentration fluctuated around about 1–3 molecules, and finally disappeared after 3.1 ps. HONO was produced at 0.7 ps, then its concentration fluctuated around about 1–2 molecules, and finally disappeared after 2.5 ps. In addition, the change in the number of OH is related to that of HONO. This also indicates that most of OH was come from HONO by the O-N bond cleavage. HNCO was formed at 1.4 ps as its structure is more stable than HONO, so HNCO lasted for a long time.

## 3. Computational Methods

Previous studies have shown that the basis set DFT-B3LYP/6-311G (d, p) could accurately predict the structure and energy of organic compounds. Therefore, the DFT-B3LYP method with the 6-311G (d, p) basis set is used to optimize molecular structure and to predict properties [6,32,33,34].

The gas phase HOF can be estimated on the basis of the isodesmic reactions, in which the number of various bonds could be kept constant to reduce the calculation error [35,36,37]. The isodesmic reactions used to obtain the gas-phase HOFs of the designed compounds were shown in Figure S5 of Supporting Information.

For an isodesmic reaction, the heat of reaction Δ*H*_298K_ at 298 K can be calculated from Equation (1).

(1) $$\Delta H_{298K}=\Delta H_{f,p}-\Delta H_{f,R}$$

where Δ*H*_f,R_ and Δ*H*_f,P_ are the HOFs of reactants and products at 298 K, respectively. At the same time, the following Formula (2) can be used to calculate Δ*H*_298K_.

(2) $$\Delta H_{298K}=\Delta E_{298K}+\Delta (PV)=\Delta E_{0}+\Delta ZPE+\Delta H_{T}+\Delta nRT$$

where Δ*E*_0_ is the total energy change between the products and the reactants at 0 K, Δ*ZPE* is the difference between the zero-point energies (*ZPE*) of the products and reactants at 0 K, and Δ*H*_T_ is thermal correction from 0 K to 298 K. The Δ(*PV*) value is *PV* work, for ideal gas reaction, it is equal to Δ*nRT*.

Generally, most energetic compounds are solid, so the calculation of explosive properties requires solid-phase HOF (Δ*H*_f,solid_) [38]. According to Hess’s law of constant heat summation [39], the gas-phase HOF (Δ*H*_f,gas_) and heat of sublimation (Δ*H*_sub_) can be used for evaluating solid-phase HOFs by the following expression (3).

(3) $$\Delta H_{f,\mathrm{solid}}=\Delta H_{f,\mathrm{gas}}-\Delta H_{\mathrm{sub}}$$

Δ*H*_sub_ can be calculated by the empirical expression suggested by Politzer et al. [24,40].

(4) $$\Delta H_{\mathrm{sub}}=aA^{2}+b{\left(\nu \sigma_{\mathrm{tot}}^{2}\right)}^{0.5}+c$$

where *A* is the surface area of the 0.001 electrons/bohr^3^ isosurface of the molecule electronic density, *ν* describes the balance between positive and negative potential on the isosurface, and *σ*_tot_^2^ is a measure of the variability of the electrostatic potential on the molecular surface. The coefficients *a*, *b*, and *c* are 2.670 × 10^−4^ kcal/mol/A^4^, 1.650 kcal/mol, and 2.966 kcal/mol, respectively [41]. The descriptors *A*, *ν*, and *σ*_tot_^2^ can be calculated using the computational procedures proposed by Bulat et al. [42]. This method has been proven to accurately estimate the heat of sublimation of many energetic compounds [43,44].

The crystal density can be obtained on the basis of the improved method proposed by Politzer et al. [45] in which the interaction index *νσ*_tot_^2^ is introduced by expression (5).

(5) $$\rho =\alpha \left(\frac{M}{V\left(0.001\right)}\right)+\beta \nu \left(\sigma_{\mathrm{tot}}^{2}\right)+\gamma \;$$

where *M* is the molecular mass (g/mol) and *V*(0.001) is the volume of the 0.001 electrons/bohr^3^ electronic density of the molecule (cm^3^/molecule). The coefficients *α*, *β*, and *γ* are 0.9183, 0.0028, and 0.0443, respectively [46].

The detonation velocity and pressure are estimated by the Kamlet–Jacobs equations [47].

(6) $$D=1.01{\left(N{\bar{M}}^{1/2}Q^{1/2}\right)}^{1/2}(1+1.30\rho )$$

(7) $$P=1.558\rho^{2}N{\bar{M}}^{1/2}Q^{1/2}$$

where each term is defined as follows: *D*, detonation velocity (km/s); *P*, detonation pressure (GPa); *N*, the number of moles of detonation gases per gram explosive; $\bar{M}$, the average molecular weight of these gases; and *Q*, heat of detonation (cal/g). According to the exothermic reaction principle, it can be evaluated by the differences of HOF between product and explosive; and *ρ*, the crystal density of explosives (g/cm^3^).

The impact sensitivity (*h*_50_, cm) can be predicted by the equation proposed by Pospíšil et al. [14,48].

(8) $${\;h}_{50}{=m\sigma }_{+}^{2}+n\nu +q$$

where *h_50_* is measured with a hammer with a mass of 2.5 kg dropping upon a sample to determine the height from which there is a 50% probability of causing an explosion [49,50]. $\sigma_{+}^{2}$ is the indicator the strengths and variability of the positive surface potentials, and *ν* is the balance of charges between positive potentials and negative potentials on the molecular surface. The coefficients *m*, *n*, and *q* are −0.0064, +241.42, and −3.43, respectively.

The Monte Carlo (MC) sampling was performed for each of the space groups using fixed molecular structures. The empirical Dreiding Force Field (FF) as implemented in Polymorph code was used to minimize the most stable structures with allowing the molecules and lattice to relax. The most possible molecular packing was predicted according to both the total energy and density.

The intermolecular interaction energy could be calculated by the energy differences between the bimolecular assemble and monomer. The BSSE was corrected by CP [51]. The intermolecular interaction energy of the bimolecular assemble can be calculated from the following Equation (9).

(9) E(int.)=E(A/A)-E(A)-E(A)+E(BSSE)

where *E*(int.) is the complex interaction energy, *E*(A/A) is the total energy of the bimolecular assemble, *E*(A) is the monomer energy, *E*(BSSE) is the CP correction energy, and the unit is kJ/mol.

In order to obtain more accurate information about hydrogen bonding interactions, a topological analysis on a variety of molecular clusters was performed using QTAIM [52]. The electron wave functions of the molecules in the clusters were calculated at the level of B3LYP/6-31G (d, p) in the Gaussian 09 package. The electron densities (*ρ*(r)), Laplacian of electron densities (∇^2^*ρ*(r)), Laplacian of kinetic energy (*G*_b_(r)), potential energy density (*V*_b_(r)), and energy density (*H*_b_(r)) are derived from the wave functions of particular cluster structures and can be used to characterize the types and strengths of bonding. In addition, the RDG method has been successfully used to rationally explain the interactions in the explosives. RDG can display the interactions between molecules and help us analyze the interactions between molecules [20]. All the QTAIM and RDG analyzed in this work were generated by Multiwfn 3.0.

AIMD method in the DFT framework was used to simulate the thermal decomposition of the R3 crystal under the CASTEP package, using Troullier–Martins norm-conserving pseudopotentials and the plane-wave expansion of the wave functions [53]. The plane wave cutoff was set to 900 eV for structure optimization and 300 eV for AIMD simulations. Sampling in the Brillouin area adopts the Monkhorst-Pack scheme, and k-point grid is 1 × 1 × 1. The values of the kinetic energy cutoff and the k-point grid were determined to ensure the convergence of total energies [54].

The molecular, crystal, and supercell structures of R3 were shown in Figure S6 of Supporting Information. The structure was optimized by using the Broyden, Fletcher, Goldfarb, and Shannon (BFGS) method and Perdew-urke-Ernzerhof (PBE) functional in generalized gradient approximation (GGA) [55]. Considering long simulation time, a 2 × 1 × 1 supercell was selected as a model for AIMD simulations, as shown in Figure S6(c) of Supporting Information. An NVT ensemble was selected, and the system temperature was controlled by a Nosé thermostat [56]. The simulation procedure is as follows. First, the dynamic simulation was performed for the optimized crystal structure at 298.15 K for 10 ps. Then, based on the equilibrium system, another simulation was performed for 20 ps with a time step of 1.0 fs at 2500 K in the same way. We outputted one frame every 5 fs and sum accounts to 4000 frames. We analyzed one frame every 200 fs. The time of 200 fs is much longer than the barrier crossing time of about 100 fs in the transition-state theory. All of the major decomposition products appeared and were sufficient for us to describe the primary and secondary chemical reactions within the total simulation time. The bond-distance cutoff criteria used to identify the individual species during decomposition were provided in Table S3 [57]. A chemical bond was considered to be formed between two atoms if the two-atom spacing was less than the bond length distance criterion within a minimum bond lifetime of 200 fs. Therefore, we can derive the reaction rule of reaction products with the time as well as the decomposition mechanisms. The adoption of this criterion can present qualitative conclusions in this work.

## 4. Conclusions

In this work, three new bicyclic and four new cage compounds were designed on the basis of the RDX system. Then, their electronic structures, HOFs, energetic properties, impact sensitivity, and intermolecular interactions in the bimolecular assembles were studied using the DFT-B3LYP method. Finally, the thermal decomposition of R3 was stimulated by AIMD.

All the designed compounds have higher densities, better detonation properties, and better oxygen balances than the parent compound RDX. Especially, they all have very excellent detonation properties. In addition, R1 and R3 show excellent detonation properties superior to RDX and HMX, comparable with CL-20, and lower impact sensitivity than RDX. Thus, all of them are promising candidates for HEDCs. 

An analysis of intermolecular interactions indicates that R3 is the most stable, followed by R1, R2, and R4–R7. The R3 crystal has four initial reaction mechanisms and two subsequent decomposition paths at high temperature. After a series of ring opening and recombination reactions, some long chains and complex carbon-rich heterocyclic rings were formed, and then gradually broke into small fragments: CO, N_2_, NO_2_, NO, HNCO, and OH.

Our design strategy that the construction of bicyclic or cage compounds based on the system of RDX by incorporating the intermolecular -N(NO_2_)-CH_2_-N(NO_2_)-, -N(NH_2_)-, -N(NO_2_)-, and -O- linkages has been verified to be a valuable approach to develop novel HEDCs with both excellent performance and low sensitivity.

## Figures and Tables

**Table 1:** Calculated heats of detonation (*Q*), densities (*ρ*), oxygen balances (OB), detonation velocities (*D*), detonation pressures (*P*), and impact sensitivities (*h*_50_) for the designed compounds.

Compound	*ρ* (g/cm^3^)	*Q* (cal/g)	*D* (km/s)	*P* (GPa)	OB ^1^	*h*_50_ (cm)
R1	1.78	2152.91	9.63	40.86	−4.52	33.16
R2	1.88	2291.20	10.20	47.33	0.00	25.27
R3	1.82	2089.75	9.65	41.67	−6.78	40.73
R4	1.94	2138.05	10.23	48.46	−4.19	17.32
R5	1.98	2019.73	10.19	48.67	7.77	13.87
R6	1.94	2223.84	10.27	48.91	0.00	13.90
R7	1.98	2006.87	10.14	48.13	4.35	19.68
RDX	1.77 (1.82 ^2^)	1559.99 (1501.00 ^3^)	8.78 (8.70 ^2^)	33.80 (34.00 ^2^)	−21.62	26.00
HMX	1.84 (1.91 ^2^)	1532.78 (1498.00 ^3^)	9.01 (9.10 ^2^)	36.58 (39.30 ^2^)	−21.62	30.00
CL-20	1.94 (2.04 ^2^)	1671.87 (1738.20 ^3^)	9.44 (9.40 ^2^)	41.33 (42.00 ^2^)	−10.96	11.94

^1^ Oxygen balance (%) for CaHbOcNd: 1600(*c*-2*a*-*b*/2)/*M*_W_; *M*_W_ = molecular weight of the designed compounds. ^2^ The experimental values were taken from Refs. [15,24]. ^3^ The calculated values were taken from Ref. [6].

**Table 2:** Topological parameters of seven bimolecular assembles at the (3, −1) critical points: calculated hydrogen bond length (*d*), electron densities (*ρ*(r)), Laplacian of electron densities (∇^2^*ρ*(r)), Laplacian of kinetic energy (*G*_b_(r)), potential energy density (*V*_b_(r)) and energy density (*H*_b_(r)).

Assemble	Interaction	*d*	*ρ*(r) × 10^2^	∇^2^*ρ*(r) × 10^2^	*G*_b_(r) × 10^2^	*V*_b_(r) × 10^2^	*H*_b_(r) × 10^3^
R1-R1	H(34)···O(17)	2.70	0.5023	2.0030	0.4067	−0.3126	0.9407
	H(30)···O(47)	2.71	0.5177	2.1224	0.4342	−0.3378	0.9640
	H(29)···O(47)	2.60	0.7653	2.7323	0.5946	−0.5061	0.8850
R2-R2	H(42)···O(9)	2.44	0.9488	3.2314	0.7340	−0.6601	0.7390
	H(18)···O(32)	2.50	0.8422	2.9014	0.6479	−0.5704	0.7749
R3-R3	H(40)···O(15)	2.50	0.8863	2.9218	0.6633	−0.5962	0.6713
	H(20)···O(36)	2.50	0.8864	2.9222	0.6634	−0.5963	0.6713
R4-R4	H(37)···O(23)	2.78	0.4044	1.7658	0.3425	−0.2436	0.9893
	H(5)···O(55)	2.78	0.4043	1.7654	0.3424	−0.2435	0.9892
R5-R5	O(44)···O(22)	3.25	0.3514	1.6855	0.3526	−0.2839	0.6875
	O(52)···O(14)	3.25	0.3513	1.6851	0.3526	−0.2838	0.6872
R6-R6	H(33)···O(21)	2.44	0.7850	2.8778	0.6371	−0.5547	0.8237
	H(28)···O(52)	2.86	0.2766	1.3043	0.2419	−0.1577	0.8421
R7-R7	H(5)···O(47)	2.86	0.3955	1.7819	0.3393	−0.2332	1.0616

**Table 3:** Intermolecular interaction energies *E*(int.) of seven bimolecular assembles.

Assembles	R1	R2	R3	R4	R5	R6	R7
*E*(int.) (kcal/mol)	−8.19	−7.02	−9.46	−4.94	−6.18	−6.16	−5.42
*E*(int.) (kJ/mol)	−34.27	−29.37	−39.58	−20.67	−25.86	−25.77	−22.68

## Data Availability

The data presented in this study are available on request from the corresponding author.

## Supplementary Materials

The following are available online. Table S1: Calculated total energy (*E*_0_), zero-point energy (*ZPE*), thermal correction (*H*_T_), and HOFs of the reference compounds. Table S2: Calculated total energy (*E*_0_), zero-point energy (*ZPE*), thermal correction (*H*_T_), gas-phase HOFs (Δ*H*_f,gas_), heat of sublimation (Δ*H*_sub_), and solid-phase HOFs (Δ*H*_f,solid_) for the designed compounds. Table S3. Bond type and bond cutoff radius in the fragment analysis of the AIMD trajectory. Figure S1: Relationship between crystal density and total energy of the packing for R1–R7. Figure S2: Optimized bimolecular structures and atom numbers. Figure S3: Snapshots of the decomposition mechanisms of (i). Figure S4: Snapshots of the decomposition mechanisms of (iii). Figure S5: The corresponding isodesmic reactions of seven designed compounds (R1−R7). Figure S6: Molecular structures of R3 (a) and a unit cell (b) and a 2 × 1 × 1 supercell (c) of R3 crystal. Gray, blue, red, and white spheres stand for carbon, nitrogen, oxygen, and hydrogen atoms, respectively.

## Sample Availability

Samples of the compounds are not available from the authors.
