# Supplementary material for: Computational Design of High Energy RDX-Based Derivatives: Property Prediction, Intermolecular Interactions, and Decomposition Mechanisms
Source: Molecules. 2021 Nov 27;26(23):7199. doi: 10.3390/molecules26237199 (PMC8659176; doi:10.3390/molecules26237199)
Supplement: Supplementary file 1 [file molecules-26-07199-s001.zip › molecules-1454703-supplementary.pdf]

Article

Computational Design of High Energy RDX-based Derivatives: Property Prediction, Intermolecular Interactions, and Decomposition Mechanisms

Li Tang and Weihua Zhu *

Institute for Computation in Molecular and Materials Science, School of Chemistry and Chemical Engineering, Nanjing University of Science and Technology, Nanjing 210094, China

* Correspondence: zhuwh@njust.edu.cn

Table S1. Calculated total energy (E_0), zero-point energy (ZPE), thermal correction (H_T), and HOFs of the reference compounds.[1.]

Compound	E_0(a.u)	ZPE(a.u) [1]	H_T(kJ/mol) [1]	HOF(kJ/mol) [2]	HOF(kJ/mol) [3]
NH_3	-56.5826	0.0342	10.05	-45.90	-45.10
NH_2NO_2	-261.1248	0.0392	12.39	-3.20	-1.90
NH_2NH_2	-111.8976	0.0527	12.23	95.40	110.66

[1] The scaling factor for ZPE was 0.98 and the scaling for H_T was 0.96 [48]. [2] The experimental values were taken from Ref. [31]. [3] The calculated values were calculated at the G2 level.

Table S2. Calculated total energy (E_0), zero-point energy (ZPE), thermal correction (H_T), gas-phase HOFs ($\Delta H_{f,gas}$), heat of sublimation (ΔH_{sub}), and solid-phase HOFs ($\Delta H_{f,solid}$) for the designed compounds.

Compound	E_0(a.u)	ZPE(a.u) [1]	H_T(kJ/mol) [2]	$\Delta H_{f,gas}$(kJ/mol)	ΔH_{sub}(kJ/mol)	$\Delta H_{f,solid}$(kJ/mol)
R1	-1455.15	0.19	56.04	1250.84	164.82	1086.01
R2	-1155.98	0.14	44.18	1118.18	98.16	1020.03
R3	-971.37	0.12	36.44	699.88	103.83	596.05
R4	-1564.62	0.20	58.74	1445.52	131.05	1314.48
R5	-1713.76	0.18	62.33	1556.46	132.49	1423.97
R6	-1453.93	0.16	52.92	1334.44	116.90	1217.54
R7	-1529.15	0.17	54.78	1150.88	118.54	1032.34

[1] The correction factor for ZPE was 0.98.

[2] The correction factor for H_T was 0.96.

Table S3. Bond type and bond cutoff radius in the fragment analysis of the AIMD trajectory.

Bond type	Cutoff radius(Å)	Bond type	Cutoff radius(Å)
C-C	2.31	H-O	1.50
C-H	1.63	H-N	1.61
C-O	2.15	O-O	2.22
C-N	2.20	O-N	2.10
H-H	1.11	N-N	2.17

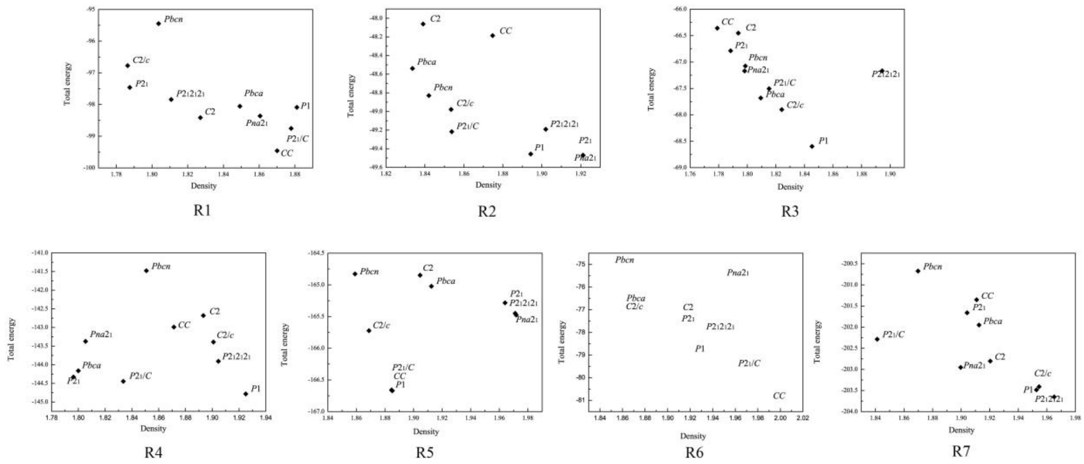

Figure S1. Relationship between crystal density and total energy of the packing for R1-R7.

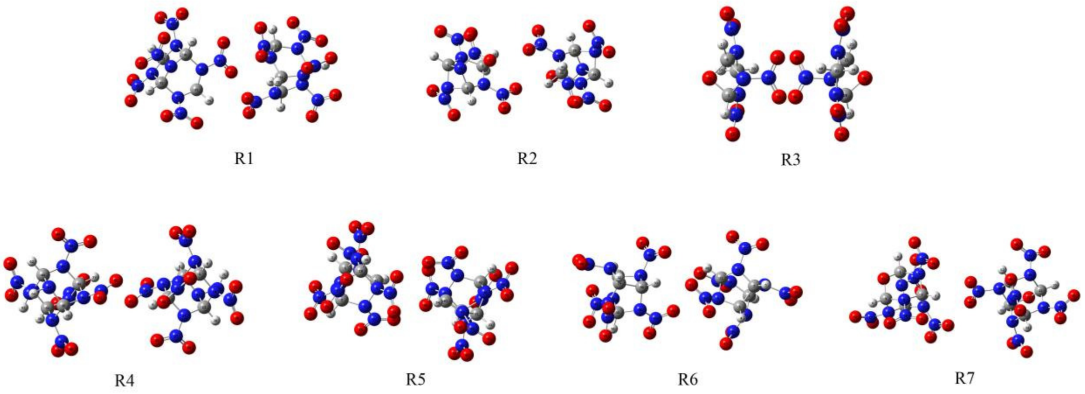

Figure S2. Optimized bimolecular structures and atom numbers.

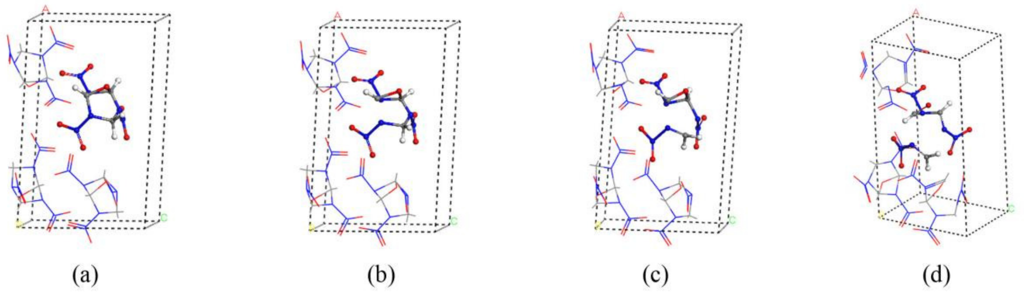

Figure S3. Snapshots of the decomposition mechanisms of (i).

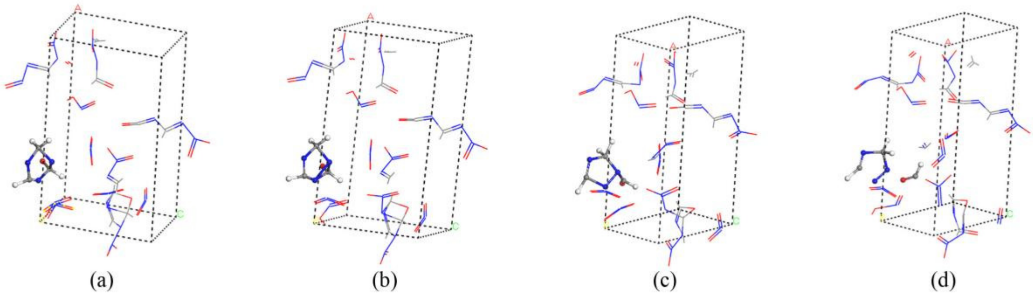

Figure S4. Snapshots of the decomposition mechanisms of (iii).

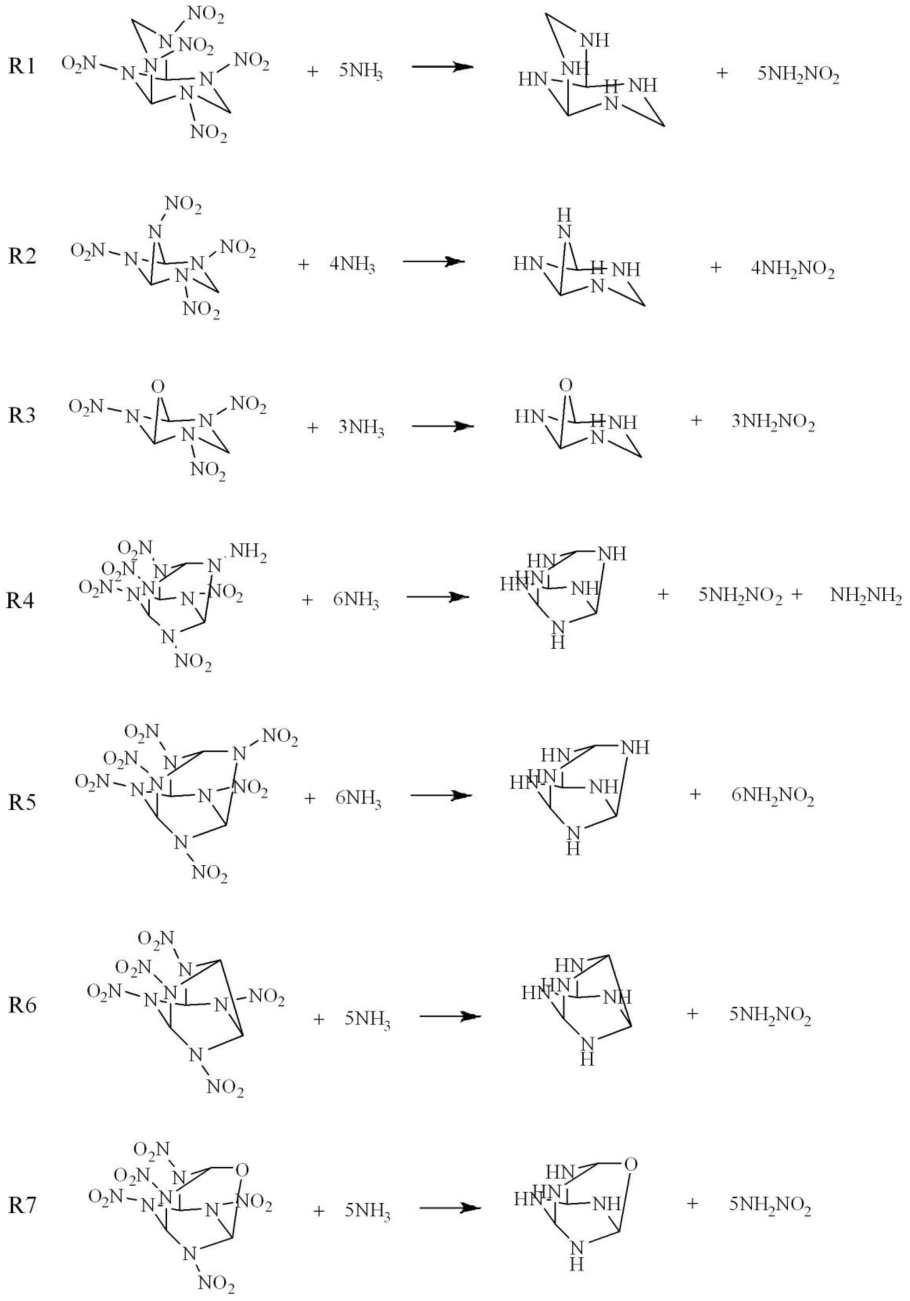

Figure S5. The corresponding isodesmic reactions of seven designed compounds (R1−R7).

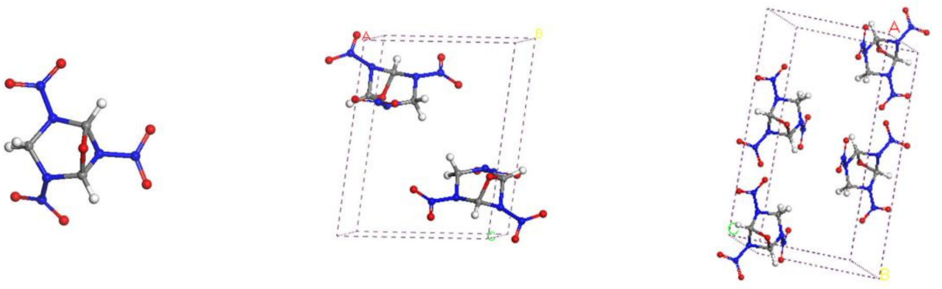

(a) (b) (c)

Figure S6. Molecular structures of R3 (a) and a unit cell (b) and a 2 × 1 × 1 supercell (c) of R3 crystal. Gray, blue, red, and white spheres stand for carbon, nitrogen, oxygen, and hydrogen atoms, respectively.
